# Gene expression analysis in human osteoblasts exposed to dexamethasone identifies altered developmental pathways as putative drivers of osteoporosis

**DOI:** 10.1186/1471-2474-8-12

**Published:** 2007-02-12

**Authors:** Conor J Hurson, Joseph S Butler, Dominic T Keating, David W Murray, Denise M Sadlier, John M O'Byrne, Peter P Doran

**Affiliations:** 1 General Clinical Research Unit, School of Medicine and Medical Sciences, University College Dublin, Mater Misericordiae University Hospital and Dublin Molecular Medicine Centre, Dublin, Ireland; 2 Department of Orthopaedic Surgery, Royal College of Surgeons in Ireland, Cappagh National, Orthopaedic Hospital, Dublin, Ireland

## Abstract

**Background:**

Osteoporosis, a disease of decreased bone mineral density represents a significant and growing burden in the western world. Aging population structure and therapeutic use of glucocorticoids have contributed in no small way to the increase in the incidence of this disease. Despite substantial investigative efforts over the last number of years the exact molecular mechanism underpinning the initiation and progression of osteoporosis remain to be elucidated. This has meant that no significant advances in therapeutic strategies have emerged, with joint replacement surgery being the mainstay of treatment.

**Methods:**

In this study we have used an integrated genomics profiling and computational biology based strategy to identify the key osteoblast genes and gene clusters whose expression is altered in response to dexamethasone exposure. Primary human osteoblasts were exposed to dexamethasone *in vitro *and microarray based transcriptome profiling completed.

**Results:**

These studies identified approximately 500 osteoblast genes whose expression was altered. Functional characterization of the transcriptome identified developmental networks as being reactivated with 106 development associated genes found to be differentially regulated. Pathway reconstruction revealed coordinate alteration of members of the WNT signaling pathway, including frizzled-2, frizzled-7, DKK1 and WNT5B, whose differential expression in this setting was confirmed by real time PCR.

**Conclusion:**

The WNT pathway is a key regulator of skeletogenesis as well as differentiation of bone cells. Reactivation of this pathway may lead to altered osteoblast activity resulting in decreased bone mineral density, the pathological hallmark of osteoporosis. The data herein lend weight to the hypothesis that alterations in developmental pathways drive the initiation and progression of osteoporosis.

## Background

Osteoporosis is a skeletal disorder characterised by low bone mass and micro-architectural deterioration with consequent increase in bone fragility and susceptibility to fracture [1]. After aging and sex steroid deficiency, the therapeutic use of glucocorticoids is the most common cause of osteoporosis. Osteoporotic fractures are an important cause of morbidity and mortality [2], particularly in elderly women who often suffer multiple fractures [3]. Indeed, approximately 40% of all white women and 13% of white men in the United States experience at least one clinically significant fragility fracture in their lifetime [4].

Glucocorticoids modify the proliferative and metabolic activity of bone cells [5-8]. They inhibit osteoblastogenesis and osteoclastogenesis and reduce osteoblast lifespan of [9-11]. These changes lead to glucocorticoid-induced osteoporosis, via reduced bone formation. Decreased bone formation has been demonstrated histomorphometrically and clinically [7]. Histomorphometric analysis showed diminished bone formation and turnover in dexamethasone-treated mice [11]. Decreases in serum osteocalcin were observed in patients given dexamethasone pulse treatment [12].

Microarray technology is one of the latest and most significant breakthroughs in experimental molecular biology [13]. The use of microarrays makes it possible to demonstrate the fundamental genes being expressed in tissues and cultured cells [14,15] Microarray technology is being used in attempts to understand fundamental aspects of growth and development, as well as to explore underlying genetic causes of many human diseases [16]. Leclerc et al have previously employed a microarray-based strategy to identify MC3T3 cell genes whose expression is altered in response to dexamethasone. These studies demonstrated the complexity of the response to steroid and the effect on specific functional families, including BMPs, extracellular matrix and signalling genes. [17]. A major limitation of these studies is the use of the mouse osteoblast like cell line MC3T3. In this study we have determined the response to dexamethasone of primary human osteoblasts, investigations that complement previously reported experiments. In this study we have utilised oligonucleotide microarrays to determine the transcriptomic response of human osteoblasts and further describe the molecular mechanisms underpinning steroid associated bone density loss.

## Methods

### Cell culture and dexamethasone exposure in vitro

Primary Human Osteoblsts were obtained from Promocell, (Heidelberg, Germany) and cultured according to the manufacturers instructions. For stimulation experiments cells were serum starved overnight. Following overnight Incubation In serum free media, 10 ng/ml dexamethasone was added to each stimulation sample at the appropriate time.

### Microarray analysis

RNA isolation, cDNA synthesis, *in vitro *transcription and microarray analysis were performed as previously reported [18]. Biotin-labelled cRNA prepared from template cDNAs was fragmented and hybridized to the Affymetrix HgU133A oligonucleotide microarrays as per Affymetrix protocol (Affymetrix, Santa Clara, CA). Arrays were then washed and fluorescently labelled prior to scanning with a confocal scanner. All *in vitro *time points were microarrayed in duplicate. Image files were obtained through Affymetrix GeneChip software (MAS5). Robust multichip analysis (RMA) was performed [19]. As each *in vivo *time-point was microarrayed in duplicate an average RMA value was computed. To ensure the average was statistically representative a t-test and p-value were generated. Only those genes with a p-value of δ 0.01 were included in subsequent bioinformatic analysis. Thereafter, expression data for each time point was compared to control and a signal log ratio of 0.6 or greater (equivalent to a fold change in expression of 1.5 or greater) was taken to identify significant differential regulation [20]. Using normalised RMA values, Unsupervised Average Linkage Hierarchical Cluster Analysis was performed [21]. A list of 1092 developmental genes represented on the Affymetrix HgU133A oligonucleotide microarray was curated via the Onto-Compare and Gene-Ontology (GO) databases [22].

### Real Time quantitative PCR

Real time RT-PCR was performed on a TaqMan ABI 7700 Sequence Detection System^® ^(AppliedBiosystems, Weiterstadt, Germany) using heat activated TaqDNA polymerase (Amplitaq Gold, Applied Biosystems, Weiterstadt, Germany), according to the manufacturers instructions. For all quantitative analyses cDNA content of each sample was compared with another sample following the ΔΔCt technique [23]. 18S rRNA and which was amplified in parallel with the genes of interest, served as housekeeping gene. Primer and probes for the genes of interest were designed in PrimerExpress^® ^(AppliedBiosystems, Weiterstad, Germany) and searched against the public databases to confirm unique amplification products. Controls consisting of dH_2_O were negative in all runs. All measurements were performed in duplicates.

## Results

### Global changes in gene expression elicited by dexamethasone

**Figure 1:**
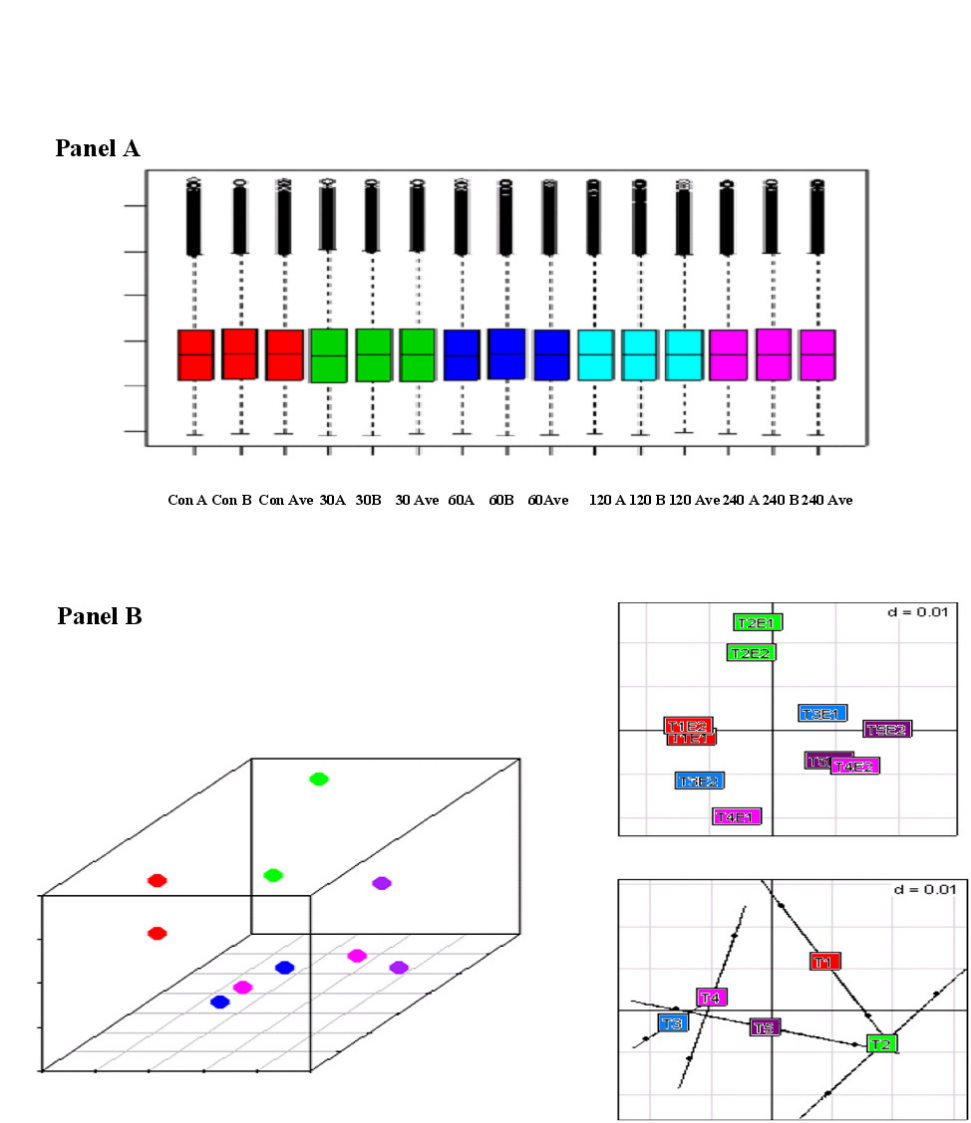
**Global Changes in osteoblast gene expression in response to dexamethasone**. Gene expression was assessed using Affymetrix HG-U133A oligonucleotide microarrays. Panel A shows boxplot of normalised data and computed average arrays for each time point. Panel B shows a principal components analysis plot of all arrays.

**Figure 2:**
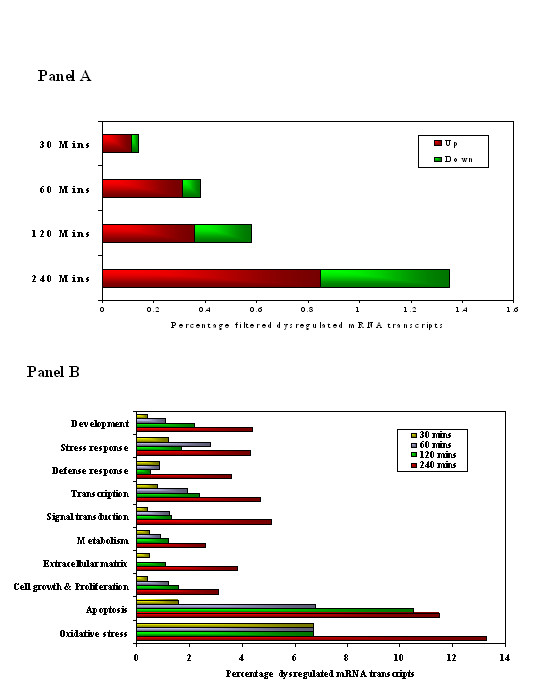
**Gene expression changes in osteoblasts exposed to dexamethasone**. Comparisons were made between control and 30, 60, 120 and 240 minute time exposures to dexamethasone. Panel A shows a bar graph representing the percentage of genes represented on the microarray that were found to be significantly altered at each time point. T1 = control, T2 = 30 mins, T3 = 60 mins, T4 = 120 mins and T5 = 240 mins dexamethasone exposure. All significantly dysregulated genes (SLR < -0.6 & SLR > 0.6) were used in classification searched. Panel B shows bar chart describing the dysregulated transcripts, from each family that were found to be significantly changed at each time point. Panel

Exposure of primary human osteoblasts to 10 ng/ml dexamethasone was associated with significant changes in gene expression. Data was normalised using RMA express and an average expression measure for each time point used to identify alterations in gene expression. RMA normalised data was found to be comparable across the time series with the computed average expression aligning to the individual chip hybridisation boxplots (Figure 1, Panel A). Principal components analysis of normalised data showed separation of the samples, demonstrating overall changes in transcriptome activity in primary human osteoblasts exposed to dexamethasone (Figure 1, Panel B). Hierarchical cluster analysis of the arrays was performed to confirm the average measurements were representative of each time point. The total number of genes altered was found to increase with time. The same temporal pattern of gene expression alterations was observed for both up and down regulated transcripts; however of note was the find that significantly more genes were upregulated in response to dexamethasone than were downregulated. Of the 22,216 gene sequences represented on the Affymetrix HGU133A oligonucleotide microarray 0.2% (31) genes), 0.47% (83 genes), 0.6% (130 genes) and 1.3%% (300 genes) were significantly altered following 30 minutes, 60 minutes, 2 hour and 4 hour exposure to dexamethasone respectively (Figure 2, Panel A).

### Ontological classification of dexamethasone-induced transcriptome identifies oxidative stress, apoptosis and developmental genes as drivers of cell injury

Having delineated the global transcriptomic response of osteoblasts to dexamethasone, we categorised the significantly perturbed genes according to their biological function. Significantly perturbed genes were used as Input In classification searches. Figure 2 Panel B shows the overall pattern of regulation of key functional families throughout the time course exposure. All families studied were found to increase over time, reflecting the increased transcriptomic activity in the latter time points. Of note with respect to the pathogenesis of steroid induced osteoporosis was the finding of major changes in apoptosis associated genes, particularly at the later time points. Increases in osteoblast apoptosis have been reported as a major pathological hallmark of osteoporosis. Other functional classes significantly represented in the dexamethasone-induced transcriptome included, extracellular matrix, cell growth and proliferation associated genes.

**Figure 3:**
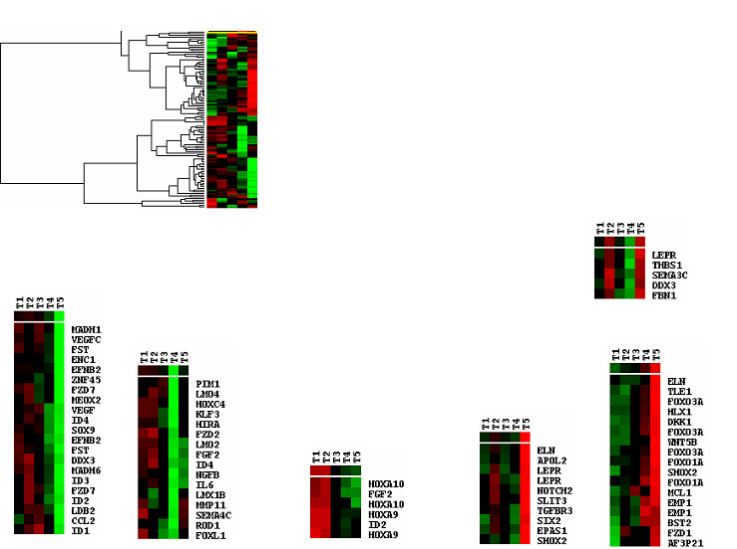
**Functional classification of dexamethasone induced transcriptome**. Hierarchical cluster analysis of developmental genes 106/1092 mRNA transcripts that encode developmental processes had a SLI of ≥ 5 and a SLR ≤ -0.4 and ≥ +0.4 in one or more timepoints compared to control (T1). This Figure shows both total cluster and specific coordinate regulation clusters of developmental genes significantly altered in at least one time point. T1 = control, T2 = 30 mins, T3 = 60 mins, T4 = 120 mins and T5 = 240 mins dexamethasone exposure

Of Interest was the large number of developmental genes that appear to be dysregulated in response to dexamethasone exposure. These data suggest that developmental processes may be subserving the cellular response in osteoporosis. Tables 1 and 2 list these developmental genes that were significantly up and downregulated in response to dexamethasone, respectively. To probe whether these developmental genes were dysregulated in a coordinated fashion hierarchical cluster analysis was performed (Figure 3). As can be seen the development-associated genes are coregulated in response to dexamethasone over the time points versus control, suggesting that the activation of developmental pathways may be a pathogenomic mechanism underpinning the altered osteoblast activity in osteoporosis.

### Development associated gene pathways are co-ordinately regulated in primary human osteoblasts in response to dexamethasone exposure

The functional classification of the dexamethasone elicited transcriptome identified developmental genes as being co-ordinately regulated. To further probe the nature of these developmental network alterations and their role in the pathogenesis of osteoporosis we determined the effect of dexamethasone on specific developmental network pathways.

Of the 1092 development associated genes represented on the Affymetrix HgU133A oligonucleotide microarrays, 106 were found to be differentially regulated (SLI of ≥ 5 and a SLR ≤ -0.4 and ≥ +0.4 in one or more time points compared to control (T1)). All 106 development classified genes identified as being significantly altered were used as input in searches of the KEGG biopathways using the Database for Annotation, Visualization and Integrated Discovery 2.0 (DAVID 2.0) resource [24]. Using this strategy 9 of the 106 development associated genes were mapped to the TGF-b1 signalling pathway; This pathway alteration included extracellular components of the (follistatin and thrombospondin 1), intracellular mediators (SMAD 1, 4, & 6) and intranuclear activators of TGF-b1 signalling (inhibitor of DNA binding 1, 2, 3 and 4).

**Figure 4:**
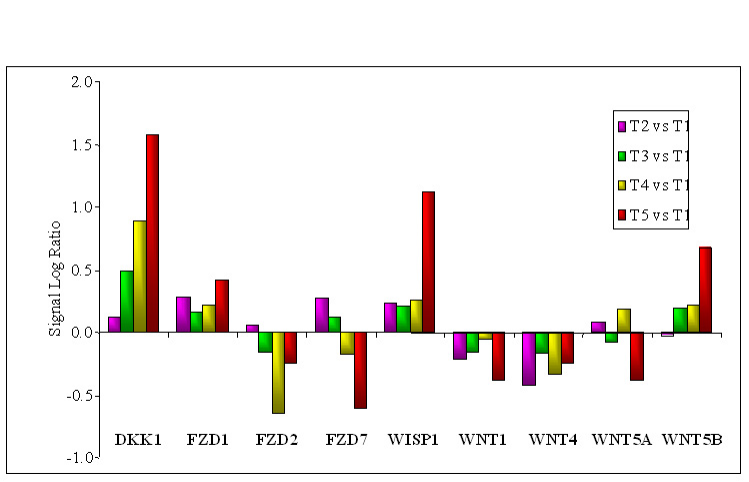
**wnt pathway genes identified as being dysregulated in osteoblasts following exposure to dexamethasone**. A list of 9 wnt pathway associated genes were identified using KEGG pathway analysis. The Microarray determined expression levels of these genes are represented in the bar chart and demonstrate the temporal regulation patterns of these genes. T1 = control, T2 = 30 mins, T3 = 60 mins, T4 = 120 mins and T5 = 240 mins dexamethasone exposure

9 of the identified development associated genes were found, using pathway analysis to be components of the WNT signalling pathway (Figure 4). The Wnt signalling pathway comprises a family of secreted glycoproteins with functions relating to cell specification, formation of the body plan, cell growth, differentiation and apoptosis. Wnt proteins bind to the frizzled family of receptors and their low-density lipoprotein-related protein (LRP) co-receptors, which transduce the signal through either the canonical β-catenin pathway or non-canonical pathway. The Wnt/β-catenin or canonical Wnt pathway is particularly important for bone biology [25,26]. Activation of this pathway occurs upon binding of Wnt to the 7-transmembrane domain-spanning frizzled receptor and LRP5/6 coreceptors. Loss of function mutations in human LRP5 are associated with osteoporosis-pseudoglioma syndrome, which causes low bone mass and skeletal fragility [27]. More direct roles for Wnt signalling in the reduction of trabecular bone formation and bone mass have been shown in studies of mice lacking the soluble Wnt inhibitor sFRP1, with the mice demonstrating reduced osteoblast and osteocyte apoptosis [28]. Current studies suggest that endogenous Wnt signalling plays an important role in osteoblatogenesis and bone formation [29].

During embryonic development, β-catenin is elevated in differentiating osteoblasts and pharmacological and genetic approaches have indicated the Wnt signalling increases bone mass through a number of mechanisms including renewal of stem cells, stimulation of preosteoblast replication, induction of osteoblastogenesis and inhibition of osteoblast and osteocyte apoptosis. β-catenin is needed for the early parts of osteoblastogenesis, as well as osteoblast maturation [30].

In human osteoblasts Wnt signalling is repressed by dexamethasone which suggests a role for this pathway in glucocorticoid induced osteoporosis [32]. Wnt 10b has been shown to increase bone mineral density throughout the weight-bearing skeleton, with increased trabecular bone in the endocortical compartment and a 4-fold increase in bone volume fraction in the distal femoral metaphysic [33,34].

**Figure 5:**
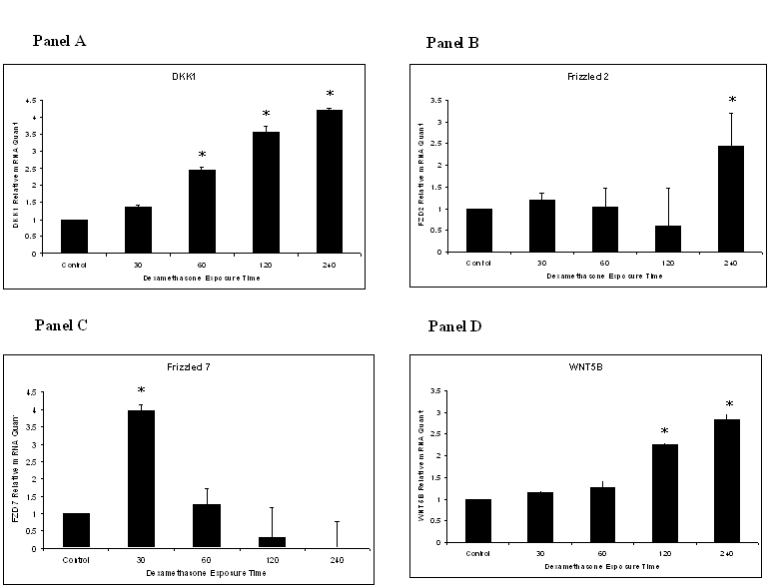
**Dexamethasone elicited alterations in developmental gene expression**. Confirmation of the microarray identified alterations in expression of DKK1, frizzled 2, frizzled 7 and WNT5B (Panels A, B, C and D respectively.) by quantitative real time PCR. All expression values were normalised to 18S rRNA. All measurements were completed in triplicate. Data are quoted relative to control. (* P < 0.05)

DKK1 is a member of the dickopff family of secreted inhibitors of wnt signalling. DKK1 has been shown to block WNT2-induced cell growth in cultured fibroblasts and the WNT2-induced increase in uncomplexed beta-catenin Of note with respect to the pathogenesis of osteoporosis, was the finding that production of DKK1, which inhibits osteoblast differentiation, is associated with the presence of osteolytic bone lesions in patients with multiple myeloma. [35]. Figure 5 Panel A demonstrates temporally regulated induction of this mRNA by dexamethasone in primary human osteoblasts, with significant increases in expression detected 60 minutes post exposure, a trend that continued for 4 hours. These data lend further weight to the hypothesis that altered developmental networks drive the pathogenesis of steroid associated bone disease.

Frizzled 7 is a member of the frizzled family of wnt protein receptors. The frizzled-dependent signalling cascade comprises several branches whose differential activation depends on specific Wnt ligands, frizzled receptor isoforms, and the cellular context. During gastrulation, frizzled-7-dependent PKC signalling controls cell-sorting behaviour in the mesoderm [36]. The activity of this mediator in the canonical wnt pathway suggests its differential regulation may be a key effector of altered osteoblast differentiation in the setting of osteoporosis. Real time PCR identified an initial increase in expression of Frzzled-7 following exposure to dexamethasone for 30 mins, with the level of expression being reduced at subsequent time points (Figure 5, Panel B)

The frizzled-2 receptor binds WNT proteins and can signal by activating calcium release from intracellular stores. Whilst its exact function in the setting of bone remains to be elucidated, recent data indicate that endogenous expression of WNT antagonists by osteoblasts controls osteoblast maturation and subsequent functional activity [37]. Figure 5 Panel C, demonstrates significantly enhanced expression of frizzled 2 following exposure to dexamethasone for four hours.

WNT5B is a member of the wnt family of genes. The WNT gene family consists of structurally related genes encoding secreted signalling molecules that have been implicated in oncogenesis and in several developmental processes, including regulation of cell fate and patterning during embryogenesis. Members of the WNT family of secreted signalling molecules have been implicated in regulating chondrocyte differentiation. WNT5B is expressed in a subpopulation of prehypertrophic chondrocytes in the developing chicken limb [38]. Furthermore this family is thought to play a key role in the regulation of osteogenesis and skeletal development. Figure 5 Panel D shows significant increases in the expression of WNT5B at both 2 and 4 hours post dexamethasone exposure.

## Discussion and conclusion

Glucocorticoids modify the proliferative and metabolic activity of bone cells. They inhibit osteoblastogenesis and osteoclastogenesis and reduce the lifespan of osteoblasts. They are also potent repressors of osteoblast function and probably stimulators of mature osteoclasts. Together, these changes lead to glucocorticoid-induced osteoporosis, mainly via reduced bone formation.

The direct impairment of the proliferative and metabolic activity of osteoblasts is mediated by a number of different mechanisms. In recent years, research in the field of glucocorticoids and bone metabolism has focused to a significant extent on the capacity of glucocorticoids to inhibit the synthesis of cytokines and their binding proteins and also of collagen and other bone matrix proteins. Of particular importance in this regard are proinflammatory cytokines, which play a significant role in the pathogenesis of various inflammatory-rheumatic diseases. Increased bone resorption and reduced bone formation have been shown to occur especially with interleukin-1 (IL-1) and also to some extent with tumour necrosis factor (TNF)-α and TNF-β [39-42]. TNFs, in particular, are known to be potent stimulators of osteoclastogenesis [43]. IL-1 and TNFs are produced by a variety of cells (e.g., T lymphocytes, monocytes, and osteoblasts) and their synthesis can be inhibited by glucocorticoids. The generalized reduction in bone density seen in patients receiving long-term glucocorticoid therapy is therefore not due to increased effects of IL-1 and TNF [41,42] In this context, it should be noted glucocorticoids also have favourable effects on bone metabolism, for instance, on the osteoporosis that occurs in the vicinity of articular surfaces in patients with rheumatoid arthritis. Glucocorticoid therapy reduces inflammatory activity and thus also reduces the loss of bone density mediated by cytokines and mediators of inflammation [42].

Glucocorticoids have been also been noted to have effects on the extracellular matrix such as their ability to reduce levels of mRNA for type I collagen and osteocalcin and to modulate levels of mRNA for osteopontin, fibronectin, β1-integrin, bone sialoprotein, and insulin-like growth factors (IGFs). Many studies have examined the effects of glucocorticoids on the metabolism of IGF-1 and IGF-2, in particular. These two peptides are of crucial importance in bone metabolism, as they act as local regulators of bone cell function [44]. They have been shown to inhibit collagenase-3 synthesis in rat bone cell cultures *in vitro *[45]. They also inhibit proliferation of osteoblasts and reduce the amount of osteoblastic type I collagen synthesis [46]. The action of IGFs on the metabolic activity of osteoblasts affects not only osteoblastic functions, but also, via modulation of osteoblast-osteoclast interactions, osteoclastic functions, and may even disturb the formation of osteoclasts. A number of studies have shown that glucocorticoids reduce the expression of IGF-1. The functions of IGF-1 and IGF-2 depend on the presence of their binding proteins, the IGFBPs (IGF binding proteins). Six different IGFBPs have now been identified, of which IGFBP-5 has been observed to increase bone cell growth [47]. Glucocorticoids modulate the insulin-like growth factor system not only directly by inhibiting IGF-1 synthesis, but also by regulating the production of IGFBPs [48-50]

In this study we have employed an integrated functional genomics and bioinformatics based strategy to identify the key genes and gene clusters whose differential expression underpins the pathogenomic response of human osteoblasts to dexamethasone. The global population age trend is increasing with the result that more patients are progressing to end stage osteoporosis, requiring joint replacement surgery. Steroids are widely used in clinical medicine. However increased, long-term use of steroid is contributing to the increased burden of osteoporosis globally. Despite significant advances in our knowledge pertaining to the mechanism of this disease process, novel therapeutic strategies have yet to emerge, placing considerable strain on surgical services.

The osteoblast is a key cell in bone biology and its role in depositing minerals contributes to overall bone density. Alterations in bone deposition by osteoblasts as well as bone resorption by osteoclasts are the key biological events in osteoporosis. Herein the effect of steroid exposure on osteoblast gene expression was determined using oligonucloeotide microarrays. Data analysis identified a cohort of genes whose expression was seen to alter in this setting. 31, 83, 130 and 300 genes were significantly altered following 30 minutes, 60 minutes, 2 hour and 4-hour exposure to dexamethasone respectively. A major limitation of microarray strategies is the large amount of uncurated data that is routinely produced. To make sense of the large amount of transcriptome information obtained, functional and pathway classification of significantly altered genes was completed. These strategies identified developmental networks as being key gene clusters altered in response to dexamethasone. Further analysis identified genes at checkpoints in the Wnt signalling pathways as being dexamethasone targets. Increasing evidence proposes a major role for the Wnt pathway in the elaboration of bone disease, as well as its physiological role in skeletal development and cellular differentiation. Further studies confirmed these gene expression changes, using quantitative PCR.

In aggregate the data presented herein lend further weight to the hypothesis that osteoporosis arises, at least in part, from alterations in the developmental control pathways in human osteoblasts. Further studies will aim to articulate the exact mechanism of dysregulation of these developmental genes, characterise the effect of altered expression on osteoblast biology with a view to further characterising these mediators as indices of disease activity, diagnostic markers and therapeutic targets in osteoporosis.

## Abbreviations

Wnt Wingless-type mmtv integration site family

DKK1 Dickkopf, xenopus, homolog of

RMA Robust Multichip Analysis

KEGG Kyoto Encyclopaedia of Genes and Genomes

TGF Transforming Growth Factor

PKC Protein Kinase C

## Competing interests

The author(s) declare that they have no competing interests.

## Authors' contributions

CH performed the majority of the studies. JB participated in study design, data interpretation and drafted the manuscript. DTK prepared RNA samples and assisted in the microarray analysis. DM designed and completed the validation studies. DS performed bioinformatics analysis of data generated. JM O'B played a major role in study design and data interpretation. PPD provided direction and oversight regarding all aspects of study design and interpretation of results. He also revised and finalised the manuscript.

## Pre-publication history

The pre-publication history for this paper can be accessed here:



## Figures and Tables

**Table 1:** Developmental genes undergoing up-regulation following exposure to dexamethasone

**Accession**	**Gene**	**SLR 30**	**SLR 60**	**SLR 120**	**SLR 240**
N25732	FOXO3A	-0.1	0.4	0.9	1.6
NM_012242.1	DKK1	0.1	0.5	0.9	1.6
NM_002015.2	FOXO1A	-0.1	0.0	0.7	1.1
NM_005842.1	SPRY2	0.3	1.6	1.1	0.9
AI572079	SNAI2	0.3	0.7	1.1	0.8
NM_003243.1	TGFBR3	0.1	0.0	-0.3	0.7
NM_030775.1	WNT5B	0.0	0.2	0.2	0.7
NM_016932.1	SIX2	0.3	0.2	0.0	0.6
BC004395.1	APOL2	0.1	0.0	0.1	0.6
NM_001554.1	CYR61	0.4	0.4	0.1	0.5
NM_005359.1	MADH4	0.6	0.1	0.1	0.5
U52914.1	LEPR	0.3	0.1	-0.2	0.5
AI951720	TLE1	-0.1	0.1	0.2	0.5
AF308601.1	NOTCH2	0.2	0.0	-0.1	0.5
M24782.1	ELN	-0.1	-0.1	0.0	0.5
M60721.1	HLX1	0.0	0.1	0.2	0.5
NM_003062.1	SLIT3	0.2	0.0	0.0	0.5
NM_001430.1	EPAS1	0.2	0.1	0.0	0.5
NM_001423.1	EMP1	0.2	0.1	0.6	0.5
NM_004335.2	BST2	0.2	0.1	0.4	0.4
NM_003244.1	TGIF	0.3	0.1	0.4	0.4
AF072872.1	FZD1	0.3	0.2	0.2	0.4
AI264196	FBN1	0.2	-0.2	-0.3	0.4
AI962897	SEMA3C	0.5	-0.1	-0.3	0.4
NM_001448.1	GPC4	0.3	-0.1	0.2	0.4
AF281859.1	SHFM3	0.3	0.2	0.3	0.4
NM_000041.1	APOE	0.1	0.3	0.3	0.4
NM_016453.1	AF3P21	0.2	0.2	0.2	0.4
NM_006884.1	SHOX2	-0.1	0.0	-0.2	0.4
NM_004472.1	FOXD1	0.2	0.4	0.0	0.3
NM_005251.1	FOXC2	-0.1	0.4	0.1	0.3
NM_012062.1	DNM1L	0.4	0.0	0.0	0.3
AI812030	THBS1	0.2	0.0	-0.4	0.3
NM_001992.2	F2R	0.1	-0.6	0.2	0.2
NM_003483.2	HMGA2	0.0	0.0	0.6	0.2
H71805	MCL1	-0.1	0.8	0.6	0.1
AW190873	ROD1	0.0	-0.2	-0.5	0.1
NM_013246.1	CLC	-0.1	0.4	-0.1	0.1
NM_005882.2	MAEA	0.4	0.0	-0.2	0.1

(SLI of ≥ 5 and a SLR ≤ -0.4 and ≥ +0.4 in one or more timepoints compared to control (T1)

**Table 2:** Developmental genes undergoing most striking ownregulation following exposure to dexamethasone

**Accession**	**Gene**	**SLR 30**	**SLR 60**	**SLR 120**	**SLR 240**
BF001670	EFNB2	0.0	-0.1	-0.2	-1.3
NM_006350.2	FST	-0.2	-0.3	-0.7	-1.2
NM_005585.1	MADH6	0.2	-0.2	-0.4	-1.1
NM_001546.1	ID4	0.3	0.1	-0.6	-1.0
NM _013409.1	FST	-0.2	-0.1	-0.3	-0.9
U16797.1	EFNB2	-0.2	-0.2	-0.7	-0.9
NM_005924.1	MEOX2	0.3	-0.2	0.0	-0.9
NM_000346.1	SOX9	-0.1	0.0	-0.5	-0.8
U41813.1	HOXA9	0.0	-0.7	-0.8	-0.8
S69738.1	CCL2	0.4	0.6	0.3	-0.8
U58111.1	VEGFC	-0.1	0.1	-0.2	-0.8
NM_018951.1	HOXA10	0.1	-0.3	-0.5	-0.7
NM_002166.1	ID2	0.4	0.0	-0.4	-0.7
NM_000600.1	IL6	0.2	0.0	-0.9	-0.6
AF022375.1	VEGF	0.1	0.2	-0.4	-0.6
AI246769	HOXA9	-0.1	-0.6	-0.7	-0.6
D13889.1	ID1	0.2	0.4	0.0	-0.6
NM_002006.1	FGF2	0.1	-0.3	-0.6	-0.6
NM_018951.1	HOXA10	0.0	-0.3	-0.3	-0.6
NM_003633.1	ENC1	0.0	0.0	-0.1	-0.6
AI333651	FZD7	0.1	-0.2	-0.1	-0.6
NM_001290.1	LDB2	0.1	0.1	-0.1	-0.4
NM_002309.2	LIF	-0.1	0.2	-0.2	-0.4
NM_002506.1	NGFB	-0.1	-0.1	-0.7	-0.4
NM_005095.1	ZNF262	0.0	0.1	0.3	-0.4
NM_001356.2	DDX3	0.1	-0.1	-0.2	-0.4
NM_020249.1	ADAMTS9	-0.4	-0.3	-0.1	-0.4
AI968085	WNT5A	0.1	-0.1	0.2	-0.4
AI819238	ID2	0.0	-0.4	-0.5	-0.4
U16153.1	ID4	0.2	-0.1	-0.5	-0.4
AI967981	ZNF45	0.0	-0.1	0.0	-0.4
U54826.1	MADH1	-0.1	0.0	-0.1	-0.4
NM_002167.1	ID3	0.2	0.0	-0.1	-0.4
NM_004333.1	BRAF	-0.4	-0.4	-0.3	-0.4
NM_005574.2	LMO2	0.0	-0.1	-0.4	-0.3
R61374	HEY1	-0.4	-0.2	-0.1	-0.3
M27968.1	FGF2	0.1	-0.1	-0.5	-0.3
NM_014240.1	LIMD1	-0.3	-0.2	-0.5	-0.3
L37882.1	FZD2	0.1	-0.2	-0.6	-0.2

(SLI of ≥ 5 and a SLR ≤ -0.4 and ≥ +0.4 in one or more time points compared to control (T1)
